# Liver Stiffness, Albuminuria and Chronic Kidney Disease in Patients with NAFLD: A Systematic Review and Meta-Analysis

**DOI:** 10.3390/biom12010105

**Published:** 2022-01-08

**Authors:** Stefano Ciardullo, Cinzia Ballabeni, Roberto Trevisan, Gianluca Perseghin

**Affiliations:** 1 Department of Medicine and Rehabilitation, Policlinico di Monza, 20900 Monza, Italy; gianluca.perseghin@policlinicodimonza.it; 2 Department of Medicine and Surgery, University of Milano Bicocca, 20900 Milan, Italy; rtrevisan@asst-pg23.it; 3 Nephrology and Dialysis, Policlinico di Monza, 20900 Monza, Italy; cinzia.ballabeni@policlinicodimonza.it; 4 Endocrinology and Diabetes Unit, ASST Papa Giovanni XXIII, 24127 Bergamo, Italy

**Keywords:** fibroscan, MAFLD, NAFLD, CKD, albuminuria, type 2 diabetes

## Abstract

An association between liver stiffness, a surrogate measure of liver fibrosis, and chronic kidney disease (CKD) in patients with nonalcoholic fatty liver disease (NAFLD) has been proposed. However, most studies were small and had low statistical power. We systematically searched PubMed-MEDLINE and Scopus from inception to August 2021 for cross-sectional or cohort studies reporting the association between liver stiffness diagnosed by vibration controlled transient elastography (VCTE) and renal dysfunction. The primary outcome was CKD, defined as a composite of urinary albumin to creatinine ratio (UACR) ≥ 30 mg/g and estimated glomerular filtration rate (eGFR) < 60 mL/min/1.73 m^2^. Measures of association from individual studies were meta-analyzed using random effects models. Of the 526 titles initially scrutinized, 7 cross-sectional studies fulfilled the criteria and were included. For CKD, risk was higher in patients with liver fibrosis assessed by VCTE, compared with patients without (n = 5 studies: OR 2.49, 95% CI 1.89–3.29; test for overall effect z = 6.475, *p* < 0.001). When increased UACR was considered as an outcome, elevated liver stiffness was associated with a significantly increased risk as well (n = 3 studies: OR 1. 98 95% CI 1.29–3.05; test for overall effect z = 3.113, *p* = 0.002). Neither analysis showed significant heterogeneity (I^2^ = 0% and I^2^ = 46.5%, respectively for the two outcomes). This meta-analysis indicates that elevated liver stiffness is associated with increased odds of kidney outcomes among patients with NAFLD. Wider use of VCTE to screen for advanced fibrosis might help identify patients at risk of end-stage renal disease.

## 1. Introduction

Nonalcoholic fatty liver disease (NAFLD) is now recognized as the most common chronic liver condition, affecting a quarter of the world adult population [1] and 37% of US adults [2]. The vast majority of patients with NAFLD will not develop cirrhosis in their lifetime, due to the low overall risk and the competing risks of dying from cardiovascular disease (CVD) and extra-hepatic cancers, which remain the most common causes of death in this population [3,4]. Nonetheless, given the number of affected individuals, NAFLD has become one of the major causes of liver cirrhosis and liver transplantation, ranking second in the US [5]. A major clinical challenge is, therefore, identifying the small number of patients with more severe forms, including nonalcoholic steatohepatitis (NASH) and, most importantly, significant/advanced liver fibrosis, the strongest predictor of future clinically relevant liver-related events [6,7].

Over the past decade, several studies have shown that NAFLD may not only impact liver-related prognosis, but also act as an independent risk factor for a series of chronic conditions including CVD [8], chronic kidney disease (CKD) [9] and extra-hepatic cancers [10]. In a recent meta-analysis including studies identifying NAFLD patients using either serum biomarkers or liver ultrasound, presence of NAFLD was associated with an increased risk of developing CKD, even after adjustment for several known risk factors [11]. However, data on the impact of liver fibrosis on renal outcomes are far more limited, in part due to the invasive nature of liver biopsy [12], the gold standard technique to diagnose NASH and stage fibrosis, and its associated bleeding risks. In this context, well performing non-invasive diagnostic methods are of great clinical importance. Among them, vibration-controlled transient elastography (VCTE) has shown promise as an accurate, rapid and validated procedure to non-invasively obtain data on both steatosis (through the controlled attenuation parameter, CAP) and fibrosis (through the liver stiffness measurement, LSM) [13]. We previously showed that significant liver fibrosis on VCTE was independently associated with albuminuria and CKD in the general US population [14]. Moreover, some smaller studies performed in patients with type 2 diabetes (T2D), a major risk factor for advanced fibrosis [15], found similar results [16,17].

To overcome limitations related to small sample size and limited statistical power, the present systematic review and meta-analysis was performed. The aim is to summarize data from observational studies conducted in adult patients with NAFLD that investigated the association between liver fibrosis assessed by VCTE and CKD, defined by increased urinary albumin to creatinine ratio (UACR), reduced estimated glomerular filtration rate (eGFR) or both.

## 2. Materials and Methods

The protocol of the present systematic review and meta-analysis was registered on PROSPERO (CRD42021274391). 

### 2.1. Data Sources and Search Strategy

We systematically searched PubMed-MEDLINE and Scopus to identify articles reporting the results of observational studies (either cross-sectional or cohort studies) published up to August 2021 investigating the association between liver stiffness and CKD. The search, designed by SC and GP, was performed by SC. The complete searching strategy is shown in Supplementary Table S1. We limited our searches to human studies without pre-defined language restrictions. Reference lists of included manuscripts and review articles were hand searched to identify additional studies not covered by the original database searches. The systematic review was performed in accordance with the Preferred Reporting Items for Systematic Reviews and Meta-Analysis (PRISMA), reported in Supplementary Table S2 [18]. Given the observational nature of the included studies, we followed the reporting items proposed by the Meta-analysis Of Observational Studies in Epidemiology (MOOSE) for the meta-analysis of these studies [19].

### 2.2. Study Selection

Only studies that met the following inclusion criteria were considered for the present systematic review and meta-analysis: (1) cross-sectional or cohort design; (2) assessment of the relationship between liver stiffness and renal dysfunction; (3) availability of a measure of association (odds ratio [OR] or hazard ratio [HR]) with 95% confidence intervals (CI) for the outcome of interest; (4) a diagnosis of liver fibrosis obtained by measurement of liver stiffness using VCTE; (5) a diagnosis of renal dysfunction based on one of the following measures: reduced eGFR (<60 mL/min/1.73 m^2^), increased UACR (≥30 mg/g), a composite of the two (referred here as CKD and considered the primary outcome). Exclusion criteria were as follows: (1) studies with different designs, editorials, congress abstracts, case reports; (2) studies that did not exclude causes of liver steatosis different than NAFLD; (3) studies that did not report a measure of association with 95% CI for the outcome of interest; (4) studies that were performed in the pediatric population.

### 2.3. Data Extraction and Quality Assessment

All titles and abstracts were independently examined by two investigators (SC and GP) and full-texts of potentially relevant papers were obtained and scrutinized separately by the same authors. We resolved discrepancies by consensus, referring back to the original articles. Information was extracted from all studies on study design, country, proportion of male participants, proportion of patients with T2D, VCTE cut-off for defining liver fibrosis, the outcome of interest and covariates included in the multivariable regression models. In case of multiple publications on the same subjects, we included only the most up-to-date and comprehensive one. Risk of bias was assessed independently by two authors (SC and GP) and discrepancies were resolved by discussion. Studies were evaluated for their quality following the Newcastle–Ottawa Scale (NOS) [20]. This scale allocates a maximum of nine points for three major domains: selection of participants (maximum of four points), comparability of study groups (maximum of two points) and ascertainment of outcomes of interest (maximum of three points). Studies that received a score of 9 stars were considered to be at low risk of bias, those that scored 7 or 8 stars to be at medium risk, and those that scored ≤6 stars to be at high risk of bias [20].

### 2.4. Data Synthesis and Statistical Analysis

ORs (or HRs) and corresponding 95% CI were considered as the measure of association of interest for each eligible study. We extracted the effect size from the statistical model reporting the maximum extent of adjustment for confounders. Adjusted measures were pooled to calculate an overall estimate of effect size. We used the random effects model following the method of Der Simonian and Laird, with the estimate of heterogeneity being taken from the Mantel–Haenszel model. Statistical heterogeneity was evaluated by visual inspection of the forest plot, as well as by the Cochrane Q test and the I^2^ statistics, which represents the proportion of the observed variability that cannot be explained by chance alone. A value of I^2^ of 0–25% represents insignificant heterogeneity, more than 25% but less than or equal to 50% represents low heterogeneity, more than 50% but less than or equal to 75% represents moderate heterogeneity, and more than 75% represents high heterogeneity [21].

A funnel plot was constructed to evaluate the presence of publication bias by plotting the logarithm of the effect measure against the logarithm of its standard error. If more than 10 studies were included in the meta-analysis, we planned to use the rank correlation Begg’s test as well [22]. Sensitivity analyses were conducted to evaluate whether the pooled effect estimate was strongly influenced by a specific study. This was performed by omitting one study each time and recalculating the pooled effect estimate on the remaining studies. All statistical analyses were performed with Stata 16.0 (Stata Corp, College Station, TX, USA). A two-tailed *p* value of <0.05 was considered significant.

## 3. Results

### 3.1. Search Results

**Figure 1:**
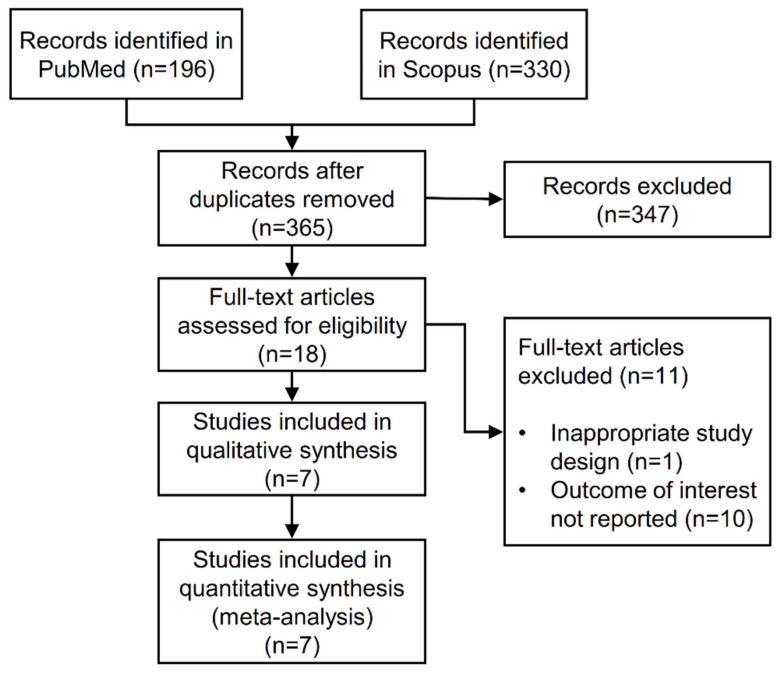
Flow diagram of study selection.

From a total of 526 articles identified by literature research, 365 were screened after duplicates were removed. Of these, 347 were excluded by reading the title and abstract based on the previously provided criteria. We examined the full text of the remaining 18 studies. After excluding articles with a different design (n = 1) or that did not report the outcome of interest (n = 10), a final number of seven included studies were analyzed and assessed for quality. A PRISMA flow diagram of the study selection is shown in Figure 1.

### 3.2. Features of the Included Articles

The main characteristics of the included studies are reported in Table 1. All were observational cross-sectional studies and most of them were performed on patients with T2D followed at university clinics. Overall, they included 7736 individuals with an age range of 42 to 69 years. Three studies were carried out in Asia (India and China), three in Europe (Italy and Croatia) and one in the US. The prevalence of T2D ranged from 20% to 100% and most studies included overweight or obese participants (mean BMI range: 27.4–34.0 kg/m^2^). All studies included a similar number of men and women (proportion of men range: 47–66%).

Four studies used a single cut-off to define significant fibrosis, while the remaining three used different cut-offs for the M and XL Fibroscan probes. Five studies reported CKD (as a composite of increased UACR and reduced eGFR) as an outcome, two reported increased UACR and one reported both. 

As shown in Table 1, six studies were considered at low risk of bias (receiving eight stars), and one was considered at medium risk (seven stars), thus indicating an overall low to medium risk of bias.

### 3.3. Association between Liver Stiffness and Kidney Outcomes

**Figure 2:**
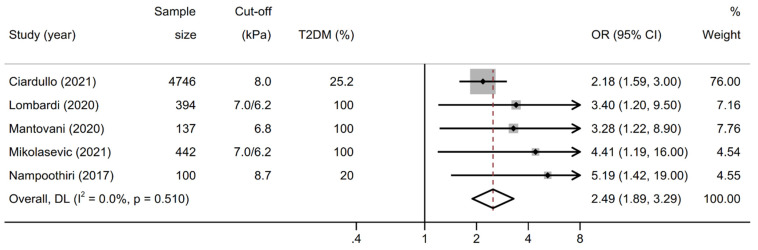
Forest plot and pooled estimates on the effect of increased liver stiffness by vibration controlled transient elastography (VCTE) on the odds of chronic kidney disease (CKD). CKD was defined as a urinary albumin to creatinine ratio ≥ 30 mg/g, an estimated glomerular filtration rate < 60 mL/min/1.73 m^2^ or both. Abbreviations: NAFLD, nonalcoholic fatty liver disease; OR, odds ratio; CI, confidence interval.

As shown in Figure 2, the pooled OR for prevalent CKD in patients with significant liver fibrosis (n = 5 studies) was 2.49 (95% CI 1.89–3.29; test for overall effect z = 6.475, *p* < 0.001) when pooling adjusted effect estimates. The test for heterogeneity was not significant (I^2^ = 0%, Cochrane’s Q = 3.29, degrees of freedom (df) = 4, *p* = 0.510). No study suggested a decreased risk of prevalent CKD in patients with significant fibrosis.

**Figure 3:**
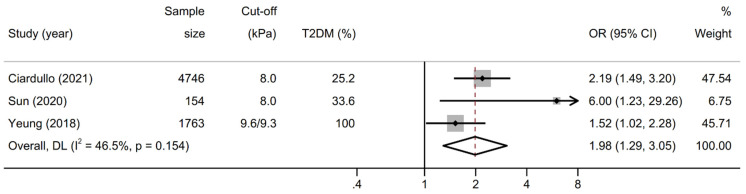
Forest plot and pooled estimates on the effect of increased liver stiffness by vibration controlled transient elastography (VCTE) on the odds of increased urinary albumin to creatinine ratio (UACR). UACR was considered increased if ≥30 mg/g. Abbreviations: NAFLD, nonalcoholic fatty liver disease; OR, odds ratio; CI, confidence interval.

As shown in Figure 3, the pooled OR for prevalent increased UACR in patients with significant liver fibrosis (n = 3 studies) was 1. 98 (95% CI 1.29–3.05; test for overall effect z = 3.113, *p* = 0.002) when pooling adjusted effect estimates. The test for heterogeneity was not significant (I^2^ = 46.5%, Cochran’s Q = 3.74, degrees of freedom (df) =2, *p* = 0.154). No study suggested a decreased risk of prevalent albuminuria in patients with significant fibrosis.

### 3.4. Sensitivity Analyses and Risk of Publication Bias

Changes in the overall effect size following omission of one study each time and recalculating the pooled effect estimate on the remaining studies are shown in Supplementary Table S3. Given the low number of included studies for each outcome, subgroup analyses were not performed. Visual inspection of the funnel plot could not exclude the possibility of publication bias (Supplementary Figure S1). As recommended by the Cochrane collaboration, tests for funnel plot asymmetry were not performed as they can be considered useful measures only when there are at least 10 studies included in the meta-analysis, because when there are fewer studies the power of the tests is too low to distinguish chance from real asymmetry [20].

## 4. Discussion

In the present meta-analysis including seven observational cross-sectional studies involving 7736 adult individuals with NAFLD from different geographical locations, we show that liver fibrosis assessed by VCTE is associated with an OR of 2.49 (95% CI 1.89–3.29) for prevalent CKD and an OR of 1.98 (1.29–3.05) for prevalent albuminuria, with no significant heterogeneity between the included studies. Moreover, most studies provided a good degree of adjustment for confounding variables including age, sex, BMI, presence of diabetes, elevated blood pressure levels or use of drugs with a proven anti-albuminuric effect (angiotensin converting enzyme inhibitors and/or angiotensin receptor blockers).

The results of the present study expand those of previous meta-analyses focusing on the association between NAFLD and incident CKD. The most recent, by Mantovani et al., which included 13 longitudinal studies, found that patients with NAFLD (diagnosed by different modalities including elevated liver enzymes, blood-based biomarkers, imaging methods and ICD-9/10 codes) had a higher risk of developing CKD (defined as an eGFR < 60 mL/min/1.73 m^2^) even after adjustment for several confounders [11]. The authors identified four studies reporting the effect of the severity of NAFLD on kidney outcomes, but since the definition of NAFLD severity was heterogeneous (based on either gamma glytamyl-transpeptidase levels, non-invasive fibrosis score or liver biopsy) no pooling of results in a meta-analysis was performed. In the present study, we focused on VCTE as the single non-invasive method included to estimate the degree of fibrosis. This was done to provide a summary of studies employing the same technique and enabled us to avoid the presence of significant heterogeneity.

It is well known that the term NAFLD comprises a large spectrum of histologic changes going from simple steatosis to advanced fibrosis and cirrhosis and that the degree of liver fibrosis represents the strongest predictor of liver-related outcomes. Current screening strategies therefore aim at identifying patients with ≥F2 fibrosis. While no international consensus exists on how to perform the screening, strategies employing VCTE or a combination of non-invasive serum-based scores (such as Fibrosis-4 or the NAFLD fibrosis score) and VCTE seem to provide the best compromise between sensitivity and risk of misclassification [27]. In this context, wider application of VCTE as a screening techniques in primary care and diabetes clinics might not only enable the identification of patients at higher risk of liver-related outcomes, but also recognize individuals that are prone to develop albuminuria and CKD, recognized risk factors for end-stage renal disease.

From a pathophysiological standpoint, several mechanisms might account for the role of liver fibrosis (and therefore stiffness) as a potential driver of albuminuria and CKD. It has been shown that NAFLD and its progression towards fibrosis are accompanied by upregulation of pro-fibrogenic cytokines (such as fibroblast growth factor-21 and transforming growth factor-β) which might act on the kidney, as well as by a low-grade inflammation and pro-thrombotic milieu [28]. Genetics might also play a role. Recent studies showed that a common polymorphism in the gene encoding the patatin-like phospholipase domain-containing protein (PNPLA3), which is strongly associated with NAFLD and progression toward fibrosis [29], seems to predispose to the development of CKD, forming a potential link between the two conditions [30]. It should also be stressed that both NAFLD and CKD represent two important risk factors for CVD, and patients with these conditions should be carefully screened for asymptomatic cardiovascular complications.

The current meta-analysis has several limitations that deserve to be acknowledged. First, given the observational nature of the included studies, it is not possible to definitely prove a causality link between the exposure and the outcome. Second, while most studies adjusted for several potential confounders including age, BMI, presence of diabetes, hypertension, use of anti-albuminuric drugs (as shown in Table 1), the possibility of residual confounding by unmeasured factors cannot be excluded. Moreover, it was not possible to combine models that accounted for the exact same variables.

Third, interpretation of our results demands cautiousness, given the cross-sectional nature of the included studies. Nonetheless, while reverse causality cannot be excluded, it is unlikely that albuminuria or CKD are involved in the pathogenesis of liver fibrosis, while the opposite cause–effect direction is suggested by previous data [31]. Future studies using a prospective design are needed to provide further evidence on the topic. Fourth, none of the included studies used a gold-standard technique, such as liver biopsy or magnetic resonance spectroscopy, to diagnose NAFLD. In fact, while these two techniques are more reliable than liver ultrasound or VCTE itself through the CAP values (which were used in the included studies), they are expensive and time consuming, making them unsuitable for large scale population studies or use in clinical practice. Moreover, liver biopsy is an invasive technique with possible (although rare) life-threatening complications, raising ethical concerns related to its use in apparently healthy subjects [12].

Our analysis also has some important strengths. It incorporates data from studies performed in Asia, Europe and the US including a representative pool of patients with NAFLD from the general population or followed at diabetes clinics. Moreover, the large number of both exposed individuals and patients with the outcome of interest yields high statistical power to quantify the association between liver stiffness and renal dysfunction, without significant heterogeneity.

## 5. Conclusions

In conclusion, this large meta-analysis shows that liver stiffness measured by VCTE is significantly associated with an OR of 2.49 (95% CI 1.89–3.29) for prevalent CKD and an OR of 1.98 (1.29–3.05) for prevalent albuminuria in patients with NAFLD. This underlies the need to carefully screen patients with NAFLD for the development of renal complications and underlies the potential benefits of a wider use of VCTE in clinical practice. Further studies with a prospective design are needed to provide more definitive evidence on the topic.

## Figures and Tables

**Table 1:** Overview of the included studies investigating the association between liver fibrosis assessed by liver stiffness and renal dysfunction in patients with nonalcoholic fatty liver disease.

Author	Year	Country	Study Design	Setting	Sample	Male (%)	Diabetes (%)	Mean Age (years)	Outcome Assessed	Adjustment	NOS Score
Ciardullo [14]	2021	USA	cross-sectional	general us population	4746	57.4	25.2	51.0	CKD, increased UACR	age, sex, race-ethnicity, BMI, diabetes, blood pressure, HbA1c, ACE-ARB therapy, CAP	8
Lombardi [17]	2020	Italy	cross-sectional	five diabetes centers	394	52.0	100.0	68.0	CKD	age, sex, smoking, diabetes duration, HbA1c, waist circumference, blood pressure, ACE-ARB therapy, statins, uric acid, LDL, HDL, insulin therapy, steatosis grade	8
Mantovani [23]	2020	Italy	cross-sectional	single diabetes center	137	48.2	100.0	69.9	CKD	age, sex, diabetes duration, HbA1c, smoking, blood pressure, dyslipidemia, BMI, HOMA-IR, hs-CRP	8
Mikolasevic [16]	2021	Croatia	cross-sectional	two diabetes centers	442	47.2	100.0	62.0	CKD	age, sex, BMI, diabetes duration, blood pressure, dyslipidemia, ACE-ARB therapy, statins, HbA1c, uric acid, hs-CRP	8
Nampoothiri [24]	2017	India	cross-sectional	single medical hospital	100	56.0	20.0	42.0	CKD	age, BMI, metabolic syndrome, HOMA-IR, transaminases, steatosis on ultrasound	7
Sun [25]	2020	China	cross-sectional	two medical centers	154	66.4	33.6	43.1	increased UACR	age, sex, ethnicity, waist circumference, uric acid, dyslipidemia, blood pressure, diabetes, HOMA-IR	8
Yeung [26]	2018	China	cross-sectional	single medical center	1763	56.0	100.0	60.7	increased UACR	age, sex, education, smoking, diabetes medications, statins, diabetes duration, dyslipidemia, HbA1c, retinopathy, blood pressure, ACE-ARB therapy, BMI	8

CKD was defined as a urinary albumin-to-creatinine ratio (UACR) ≥ 30 mg/g, an estimated glomerular filtration rate (eGFR) < 60 mL/min/1.73 m^2^ or both. Abbreviations: BMI, body mass index; HOMA-IR, homeostatic model of insulin resistance; FPG, fasting plasma glucose; HDL-C, high density lipoprotein cholesterol; TG, triglycerides; LDL, low density lipoprotein; NA, not available; HbA1c, Hemoglobin A1c; ACE, angiotensin convertin enzyme; ARB, angiotensin receptor blocker; NOS, Newastle Ottawa Scale; hs-CRP, high sensitivity C reactive protein.

## Data Availability

The dataset used for this meta-analysis is available from the corresponding author upon reasonable request.

## Supplementary Materials

The following are available online at https://www.mdpi.com/article/10.3390/biom12010105/s1, Figure S1: Funnel plot of selected studies for the primary outcome describing the relationship between effect size and standard error on the log scale. The vertical line represents the pooled effect size and the dashed lines represent the pseudo 95% confidence intervals, Table S1: Focused search strategy in MEDLINE database, Table S2: PRISMA checklist, Table S3: Sensitivity analysis of selected studies. Each row shows the recalculated pooled OR of remaining studies by omitting studies listed in the left column one at a time.
